# Micro-Raman Spectroscopy for Monitoring of Deposition Quality of High-k Stack Protective Layer onto Nanowire FET Chips for Highly Sensitive miRNA Detection

**DOI:** 10.3390/bios8030072

**Published:** 2018-07-27

**Authors:** Kristina A. Malsagova, Tatyana O. Pleshakova, Andrey F. Kozlov, Ivan D. Shumov, Mikhail A. Ilnitskii, Andrew V. Miakonkikh, Vladimir P. Popov, Konstantin V. Rudenko, Alexander V. Glukhov, Igor N. Kupriyanov, Nina D. Ivanova, Alexander E. Rogozhin, Alexander I. Archakov, Yuri D. Ivanov

**Affiliations:** 1 Institute of Biomedical Chemistry (IBMC), Moscow 119121, Russia; f17-1086@yandex.ru (K.A.M.); t.pleshakova1@gmail.com (T.O.P.); afkozlow@mail.ru (A.F.K.); shum230988@mail.ru (I.D.S.); inst@ibmc.msk.ru (A.I.A.); 2 Rzhanov Institute of Semiconductor Physics, Siberian Branch of Russian Academy of Sciences, Novosibirsk 630090, Russia; ilnitsky@isp.nsc.ru (M.A.I.); popov@isp.nsc.ru (V.P.P.); 3 Institute of Physics and Technology of Russian Academy of Sciences, Moscow 117218, Russia; motoknerva@gmail.com (A.V.M.); rudenko@ftian.ru (K.V.R.); rogozhin@ftian.ru (A.E.R.); 4 Joint-Stock Company “Novosibirsk Plant of Semiconductor Devices & DC”, Novosibirsk 630082, Russia; gluhov@nzpp.ru; 5 Sobolev Institute of Geology and Mineralogy, Siberian Branch of Russian Academy of Sciences, Novosibirsk 630090, Russia; spectra@igm.nsc.ru; 6 Skryabin Moscow State Academy of Veterinary Medicine and Biotechnology, Moscow 109472, Russia; ninaivan1972@gmail.com

**Keywords:** silicon-on-insulator, high-k dielectric, nanowire biosensor, micro-Raman spectroscopy, miRNA

## Abstract

Application of micro-Raman spectroscopy for the monitoring of quality of high-k (h-k) dielectric protective layer deposition onto the surface of a nanowire (NW) chip has been demonstrated. A NW chip based on silicon-on-insulator (SOI) structures, protected with a layer of high-k dielectric ((h-k)-SOI-NW chip), has been employed for highly sensitive detection of microRNA (miRNA) associated with oncological diseases. The protective dielectric included a 2-nm-thick Al_2_O_3_ surface layer and a 8-nm-thick HfO_2_ layer, deposited onto a silicon SOI-NW chip. Such a chip had increased time stability upon operation in solution, as compared with an unprotected SOI-NW chip with native oxide. The (h-k)-SOI-NW biosensor has been employed for the detection of DNA oligonucleotide (oDNA), which is a synthetic analogue of miRNA-21 associated with oncological diseases. To provide biospecificity of the detection, the surface of (h-k)-SOI-NW chip was modified with oligonucleotide probe molecules (oDVA probes) complementary to the sequence of the target biomolecule. Concentration sensitivity of the (h-k)-SOI-NW biosensor at the level of *DL*~10^−16^ M has been demonstrated.

## 1. Introduction

Raman spectroscopy finds its application in various forms of research, which aim at the development of biosensor technologies for the diagnosis of oncological diseases. Raman spectroscopy can be used well for the direct diagnostics of cancer pathologies [1], as well as a reference method in the development of biosensor devices [2]. With respect to serological diagnostics, there is a problem related to insufficient sensitivity of detection of the disease markers. That is, at an early stage of cancer, when the concentration of marker biomolecules in the serum is low (~10^−15^ M) [3], their revelation by direct optical label-free methods is extremely difficult. At the same time, the application of Raman spectroscopy in the development of highly sensitive nanotechnology-based diagnostic devices is promising. The urgency of development of highly sensitive next-generation devices for oncological diagnosticums is determined by the following. In 2012, ~14 million new cases of cancer were reported [4]; in 2015, the number of deaths from cancer was 8.8 million [5]. Oncological diseases are the cause of every sixth death in the world [5]. Oncological diseases cause significant economic loss, since most of the cost of treatment and rehabilitation of patients falls on state structures [6,7]. It is to be noted that therapy is much more effective at early stages of pathology development (up to 95% of positive outcomes) in comparison with terminal stages. However, due to the lack of clinically available diagnosticums for the early (asymptomatic) stage of cancer pathologies, in the majority of cases patients, however, seek medical care at later stages of the disease development [5]. At present, methods of cancer diagnosis based on the detection of protein markers are imperfect, since these proteins are, in fact, associated with inflammatory processes. At the same time, methods of molecular diagnostics based on the detection of cancer-associated miRNAs are considered to be promising [8]. Since the concentration of miRNA in blood can be at the level of 10^−15^ M and lower [9], the concentration sensitivity of miRNA detection must be very high. Currently technologies employed for miRNA detection are based on polymerase chain reaction (PCR). These methods have a number of disadvantages, one of which is the difficulty in implementation of such technologies due to the use of short probes [10]. In addition, due to the use of amplification, PCR-based methods are, unfortunately, very sensitive to contamination. This is another significant disadvantage of these methods, as it leads to false-positive results. For this reason, novel techniques for the diagnosis of cancer at an early stage are required. Thus, the development of molecular detectors that allow one to detect biological macromolecules at the single molecule level, do not require amplification and are devoid of the aforementioned disadvantages, is a high-potential direction of research.

Molecular detectors operate in the mode of counting of single macromolecules and, therefore, do not require amplification of analyte molecules for the detection of miRNA at ultra-low concentrations. This was demonstrated with the example of nanowire (NW) biosensors [11,12,13], atomic force microscope (AFM)-based detectors [14,15], nanoelectromechanical detectors [16]. Nanowire (NW) biosensors allow one to perform label-free detection of biological macromolecules in real time with high sensitivity (at femtomolar and even subfemtomolar level) and, hence, represent a promising basis for the development of novel analytical systems.

Among nanowire biosensors, one should, in turn, single out biosensors based on silicon-on-insulator structures (SOI-NW), as their fabrication is based on standard technological procedures. The principle of NW biosensor operation is based on the registration of a modulation of current flowing through the NW structure upon adsorption of analyte molecules onto its surface [11,12,17,18]. High sensitivity of NW biosensor is conditioned by high surface-to-volume ratio [19], defining the development of SOI-NW chips with smallest dimensions of sensor elements to be a relevant task. Theoretical detection limit achievable with such a biosensor can reach the level of single molecules [20]. To date, however, insufficient time stability upon operation in physiological solutions remains one of disadvantages of SOI-NW chips with native oxide. This instability is particularly critical in the case of SOI-NWs with a linear dimension of <1 μm. Earlier, we demonstrated the instability in operation of “narrow” SOI-NW structures of 90 nm width [13]. To overcome limitations caused by this disadvantage, we have developed SOI-NW structures coated with a protective layer of a dielectric based on hafnium dioxide (HfO_2_). At present, there is a great need for a technology of fabrication of high-speed silicon nanotransistors with a hafnium dioxide layer, as it allows one to reduce leakage currents and to provide high mutual conductance of the nanotransistors and, accordingly, to increase sensitivity of the biosensors employing such nanotransistors due to the higher surface potential transfer by HfO_2_ dielectrics with high-k constant (ε = 25) to its channels [21]. Increasing the h-k dielectric thickness leads to the increase in corrosion stability of HfO_2_ in water and to the decrease in leakage currents, but, at the same time, to worse sensitivity of the sensors. Decreasing the HfO_2_ thickness by changing the part of HfO_2_ layer to a more stable thin Al_2_O_3_ layer with lower ε = 9–11 is a promising way to increase the stability and sensitivity of NW sensors. Moreover, such a combined dielectric stack exhibits lower built-in charge at the interfaces with silicon [22]. Besides, technologies of mass production of SOI-NW structures with protective layers of h-k dielectrics are being intensively developed. Since these layers are very thin (<10 nm), there is a problem related with the monitoring of deposition quality of HfO_2_ layers.

In the present study, micro-Raman spectroscopy has been employed for monitoring the formation processes and stability of HfO_2_ layers on the SOI-NW chip surface. This has allowed us to control the properties of the protective HfO_2_ layer, which has been formed by plasma-enhanced atomic layer deposition (PEALD) at a deposition temperature of 300 °С and an annealing temperature from 450 to 1100 °С.

In our present study, (h-k)-SOI-NW structures with <1 μm dimensions (namely, 250 nm wide × 30 nm thick) have been fabricated. Sensor chips based on these structures have been employed for the detection of synthetic DNA analogue of miRNA-21, which is associated with several types of cancer pathologies: ovarian cancer [23], cervical carcinoma [24], glioblastoma [8,25], gastric cancer [26], non-small cell lung cancer [27], breast cancer [28,29]. To provide biospecificity of the detection, the surface of a (h-k)-SOI-NW chip was modified with oligonucleotide probe molecules (oDVA probes), which are known to be complementary to the sequence of the target molecule. It was demonstrated that such a (h-k)-SOI-NW-based biosensor with immobilized oDVA probes can be used well in the detection of their complementary oDVAs with high concentration sensitivity (detection limit (*DL*) ~10^−16^ M).

## 2. Materials and Methods

### 2.1. Chemicals

3,3′-dithiobis(sulfosuccinimidyl propionate) (DTSSP) was purchased from Pierce (Waltham, MA, USA). Potassium phosphate monobasic (KH_2_PO_4_), dimethyl sulfoxide (DMSO) and 3-aminopropyltriethoxysilane (APTES) were purchased from Sigma-Aldrich (St. Louis, MO, USA). Methanol (CH_3_OH) was from Sigma (St. Louis, MO, USA). Hydrogen peroxide (H_2_O_2_) and ethanol (С_2_Н_5_ОН, 96%) were purchased from Reakhim (Moscow, Russia). Deionized water was obtained using Milli-Q system (Millipore, Burlington, MA, USA).

### 2.2. Oligonucleotides

For the modification of the surface of working (h-k)-SOI-NW sensors, oDNA probe 5′-NH_2_-(T)_10_-ACAGCCCATCGACTGGTGTTG (Evrogen, Russia); which is complementary to the target oDNA (CAACACCAGTCGATGGGCTGT) and whose sequence corresponds to hsa-miR-21-3p [30], has been used. The surface of control (h-k)-SOINWs contained no immobilized oligonucleotides.

### 2.3. Fabrication of (h-k)-SOI-NW-Based Sensors with a Protective High-k Dielectric

NW chips were fabricated by nanostructuring using SOI-NW structures with p-type conductance. The cut-off layer thickness was 30 nm, and the buried oxide (BOX) thickness was 200 nm. Each SOI-NW has the following dimensions: width 250 nm, thickness 45 nm, length 14 μm, and the number of (h-k)-SOI-NWs on the chip was 12 [17,31,32]. Metal wiring and transistors were entirely covered with a stack of h-k PEALD dielectrics—8 nm of HfO_2_ and 4 nm of Al_2_O_3_, which provided electrical isolation and retention of sensor sensitivity in buffer solution.

The dielectric layer was deposited onto the open surface of channels of the silicon nanowire transistors with SiO_2_ native oxide, fabricated on SOI structures using the technology described in previous papers [17,32]. The contact of aluminium with р-type silicon represents a Schottky barrier with a height of up to 0.3 V. Since the chips with Schottky barriers (SB field-effect transistors (FETs)) were devoid of a protective dielectric, they were entirely coated with a h-k dielectric by PEALD using the FlexAl reactor (Oxford Instruments plc, Abingdon, Oxfordshire, UK).

Prior to dielectric deposition, immediately before PEALD procedure, the surface of SOI-NW sensors was cleaned first in H_2_O_2_/NH_3_ solution, and then in NH_3_ plasma at 500 W power and 50 mTorr working pressure for 2 min. Deposition of HfO_2_ and Al_2_O_3_ was carried out by cycles in oxygen plasma. 8-nm-thick HfO_2_ layer was formed from tetrakis(ethylmethylamino)hafnium(IV) precursor at 270 °С. 4-nm-thick Al_2_O_3_ layer was formed from trimethylaluminum precursor at 300 °С. After the PEALD procedure, SOI-NW sensors were annealed in the forming gas (N_2_:H_2_ = 95:5) at 425 °С and 200 mTorr for 30 min. After that, a deposition quality of HfO_2_ layers was monitored by micro-Raman spectroscopy.

PEALD allows one to obtain a stable passivation coating with excellent moisture permeation barrier properties [33]. Continuous, conformal and pinhole-free films of sub-10 nm thickness can be produced only by atomic layer deposition (ALD). One PEALD cycle consists of four steps (first precursor dosing, purge, plasma precursor, and purge), which form one monolayer of deposited film by sequential, self-limiting surface reaction.

To obtain the micro-Raman spectra, a Horiba Jobin-Yvon LabRam HR800 Raman spectrometer (HORIBA, Kyoto, Japan) was used to identify inclusions and phase compositions of the protective h-k dielectrics, as well as for their evaluation and assessment of the degree of crystallinity of the h-k coatings before and after thermal treatment and electrochemical reactions during their modifications in biological fluids. The presence of a built-in Olympus BX41 microscope (Olympus Corp., Tokyo, Japan; objective focal distance 1 mm; N.A. = 0.65; analyzed area diameter < 3 µm) allowed us to obtain information from the submicrometer areas of the channels of FETs with the coating. Confocal optical scheme allowed us to achieve maximum degree of detail (what is necessary due to small dimensions of the sensor elements) while maintaining a high speed of image acquisition when excited by focused to 1 µm beams of copper vapour laser (325 nm, 0.1 mW) or fiber-optic laser (532.1 nm, 0.1–1.0 mW) to prevent heating of the coated nanosensor surface; confocal scheme allowed us to measure the properties of the dielectric layer directly on silicon. Two laser wavelengths allow one to observe dispersion of Raman peaks, as well as to separate them from luminescence peaks. Moreover, the ultraviolet (UV) laser has a high extinction coefficient, what is important for Raman signal excitation in the case of ultrathin films.

### 2.4. Covalent Immobilization of Oligonucleotide Probes

Preliminary treatment and modification of the sensor chip surface with APTES was carried out analogously to the techniques described elsewhere [31,32]. oDVA probes were covalently immobilized onto the (h-k)-SOI-NW sensor surface using DTSSP cross-linker as described elsewhere [32].

### 2.5. Electrical Measurements

Electrical measurements were carried out using Keithley 6487 picoampermeter (Keithley Instruments Inc., Solon, OH, USA). During the measurements, the support of SOI structures was used as a control electrode (transistor gate). The dependencies of drain–source current on gate voltage *I_ds_*(*V_g_*) were obtained at *V_g_* from 0 to −60 V and *V_d_* = −0.2 V. Time dependencies of the current *I_ds_*(*t*) were recorded in real time at *V_g_* = −50 V and *V_d_* = −0.2 V.

### 2.6. Measurements with Silicon-on-Insulator Nanowire ((h-k)-SOI-NW) Biosensor

In the (h-k)-SOI-NW-based biosensor, a 500-μL measuring cell was employed, and the sensor chip with (h-k)-SOI-NW structures served as its bottom. The diameter of the sensor area was ~2 mm. The cell was equipped with a stirrer. The stirring rate was 3000 rpm.

**Figure 1:**
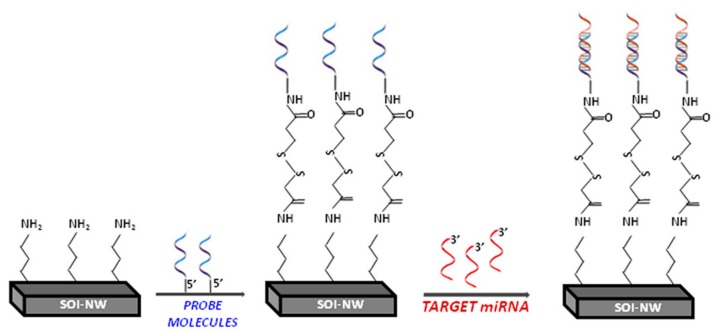
Schematic illustrating the detection of miRNA with the silicon-on-insulator nanowire ((h-k)-SOI-NW) biosensor.

For the detection of oDVA in buffer, the following registration scheme has been used. The solution to be analyzed (150 μL in 1 mM potassium phosphate buffer) was added into the measuring cell containing 300 μL of buffer, so that the final oDVA concentrations in the cell were 3.3 × 10^−14^ M, 3.3 × 10^−15^ M, 3.3 × 10^−16^ M and 3.3 × 10^−17^ M. Control experiments were carried out in the same conditions, but buffer solution without target oligonucleotide was added into the cell. Figure 1 presents a schematic illustrating the detection of miRNA with the (h-k)-SOI-NW biosensor.

To account for non-specific adsorption of target molecules, a pair of control sensors, whose surface was not sensibilized with probe molecules, was also present on the (h-k)-SOI-NW chip. The results obtained were presented in the form of time dependencies of differential signal Δ*I_ds_*(*t*). Δ*I_ds_* values are presented as log10 (Δ*I_ds_*, A) and represent the difference between the signal obtained from working (with immobilized oDNA probes) and that obtained from control (without oDNA probes) (h-k)-SOI-NW sensor. To avoid the influence of Debye screening, the detection of oDVA and miRNA was carried out in buffer with low salt concentration (1 mM potassium phosphate buffer). At this concentration of buffer salt, the Debye length (λ_D_) makes up ~5 nm [34]; this is sufficient for the registration of biomacromolecular complexes’ formation on the surface of (h-k)-SOI-NW sensor structures.

## 3. Results

### 3.1. Monitoring of Deposition Quality of HfO_2_ Layers by Micro-Raman Spectroscopy

**Figure 2:**
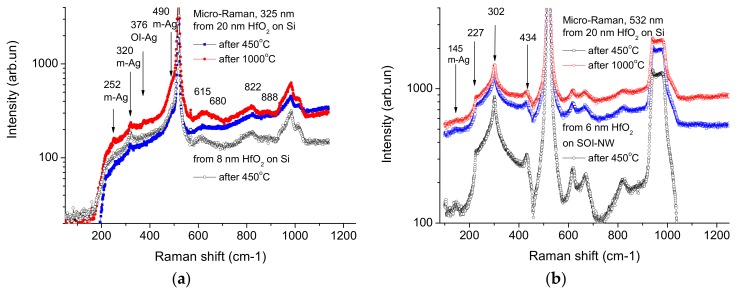
Typical micro-Raman spectra excited by laser radiation in ultraviolet (UV) (325 nm) (**a**) and visible (532 nm) (**b**) ranges on silicon wafers and SOI-NW chips. Experiment conditions: annealing temperature 450 °C (black and blue lines) and 1000 °C (red lines), HfO_2_ layer thickness 20 nm (red and blue lines) and <10 nm (black lines). Arrows indicate spectrum lines corresponding to monoclinic (m) and orthorhombic (OI) phase phonons of HfO_2_ (**a**) and acoustic phonons of Si (**b**) according to [35,36,37]).

**Figure 3:**
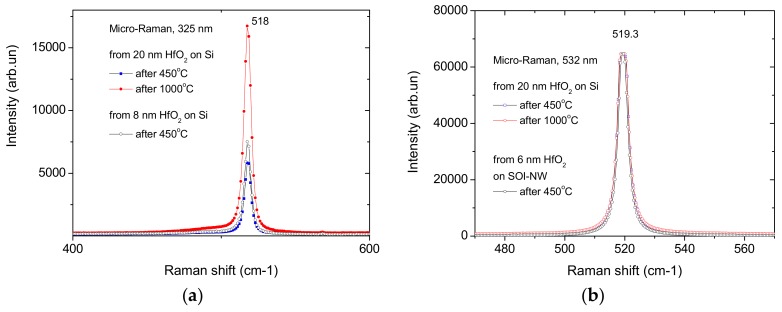
Micro-Raman spectra of single-phonon scattering peak on HfO_2_-coated silicon excited by laser radiation in UV (325 nm) (**a**) and visible (532 nm) (**b**) ranges wafers and SOI-NW chips. Experiment conditions: annealing temperature 450 °C (black and blue lines) and 1000 °C (red lines), HfO_2_ layer thickness 20 nm (red and blue lines) and <10 nm (black lines).

The monitoring of deposition quality of HfO_2_ layers was carried out by micro-Raman spectroscopy. The spectra were excited by laser radiation in UV (325 nm) and visible (532 nm) ranges. Figure 2 and Figure 3 display examples of so-obtained micro-Raman spectra.

As seen from Figure 2, the initial spectra obtained after the deposition at 300 °С do not contain any features, which could allow one to identify hafnium dioxide, except for the single-phonon scattering peak in silicon (~520 cm^−1^). After annealing at 425 °С for 30 min in the forming gas (used for firing Al contacts), appearance of several peaks in the wave vector range from 200 to 500 cm^−1^ upon excitation with UV laser, and near 145 cm^−1^ upon excitation with green laser was observed. Upon the latter excitation type, in contrast to the UV one, no other peaks were registered due to the presence of an h-k stack on the surface, on account of low absorbance even at 20 nm HfO_2_ thickness. Neither chemical treatment used for the functionalization of the h-k dielectric surface, nor subsequent washing in biologically active solutions changed the intensity of micro-Raman signals attributed to HfO_2_ crystallites. In order to increase the intensity of these signals by increasing the size and proportion of HfO_2_ crystalline phases, high temperature annealings at temperatures of up to 1100 °С have been carried out. Inthat, the increase of peaks’ intensity in the range from 200 to 500 cm^−1^ after the annealings did not exceed 30%; this is possibly connected with the annealing at 425 °С, which provided the formation of ultra-dispersed nanocrystals of HfO_2_ with sizes smaller than the h-k dielectric thickness (10 nm) (Figure 2). The high density of such nanocrystals (~70% of the volume of the h-k layer) hinders their further growth at higher annealing temperatures, also providing stability of properties of the h-k dielectrics stack in chemically and biologically active media.

The intensity of single-phonon scattering peaks (518–519 cm^−1^) in the silicon channel of SOI-NW FETs exhibits controversial behavior upon excitation with the UV (325 nm) laser. Peak intensity was expected to drop down with increasing HfO_2_ thickness, and it really exhibits such behavior for 8 nm and 20 nm films after annealing at 450 °C (Figure 3a). At the same time, increasing the annealing temperature to 1000 °C leads to the increase in the peak intensity after removing the UV light-absorbing defects and crystallization of amorphous HfO_2_ layers [38]. No dependence of peak intensity on HfO_2_ thickness was observed for green light excitation due to its low absorption in HfO_2_, and thin silicon and silica layers of SOI-NW FETs (Figure 3b). In the latter case, silicon substrate is the main absorber and light scatter.

### 3.2. Comparison of Stability in Operation of SOI-NW Chips and (h-k)-SOI-NW Chips

**Figure 4:**
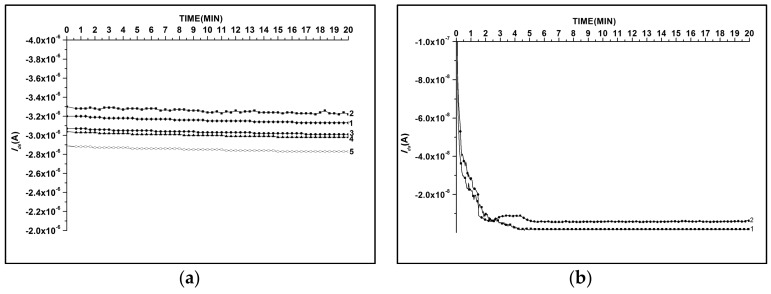
Comparison of typical *I_ds_*(*t*) dependencies obtained for (h-k)-SOI-NW chip (**a**) and for SOI-NW chip with native oxide (**b**). Numbers indicate curves obtained for different sensors. Experiment conditions: 1 mM potassium phosphate buffer (pH 7.4), *V_g_* = −50 V, *V_ds_* = −0.2 V.

The time stability of the NW chips with native oxide (SOI-NW) was compared with that of the chips bearing a protective dielectric layer ((h-k)-SOI-NW). Figure 4 displays time stability of the signal from these chips upon their incubation in 1 mM potassium phosphate buffer (pH 7.4).

The obtained results have demonstrated that in 1 mM potassium phosphate buffer the level of *I*_ds_ signal from (h-k)-SOI-NW sensors has only altered by 10% during ~8 h experiment; that is, the time stability of these chips during the experiment was 90% (Figure 4a). The time stability of SOI-NW chips (coated with only native oxide) was significantly lower. These chips often exhibited the decrease in *I*_ds_ by one to three orders of magnitude, or even a complete reduction of conductivity down to the noise level (~10^−12^ A) during the time interval required for the experiment (Figure 4b).

The results obtained allow us to conclude that the chips coated with h-k dielectrics exhibit better time stability upon their operation in NW biosensors. These chips are, therefore, preferable for use in experiments involving biological macromolecules.

After that, experiments on the detection of oDVA, which is a synthetic analogue of miRNA-21, with (h-k)-SOI-NW chips have been carried out.

### 3.3. Biospecific Detection of oDNA in Buffer

**Figure 5:**
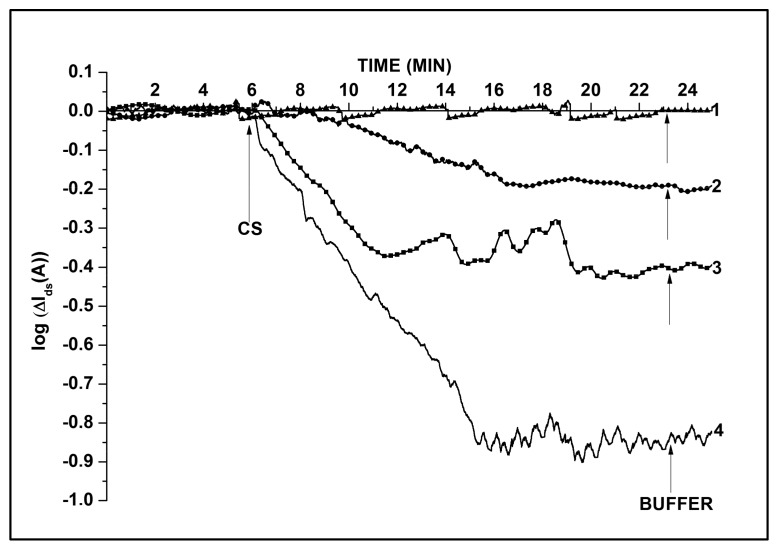
Typical Δ*I_ds_*(*t*) dependencies obtained upon detection of oDVA in buffer using (h-k)-SOI-NW sensors with immobilized oDNA probes. Experimental conditions: 1 mM potassium phosphate buffer (pH 7.4), *V_g_* = −50 V, *V_d_* = −0.2) V, total volume of solution in the cell 450 μL. Final concentrations of target oDVA in the measuring cell were 3.3 × 10^−17^ M (curve 1), 3.3 × 10^−16^ M (curve 2), 3.3 × 10^−15^ M (curve 3), 3.3 × 10^−14^ M (curve 4). Arrows indicate the addition of target oDNA solution (CS) and wash with buffer.

Figure 5 displays typical time dependencies of the current recorded before and after addition of the analyzed oDNA solutions (with concentrations from 3.3 × 10^−17^ M to 3.3 × 10^−14^ M) into the measuring cell of the biosensor. As seen from this figure, the expected increase in (h-k)-SOI-NW conductivity was observed due to adsorption of negatively charged target molecules onto the sensor surface. Also, the conductivity of (h-k)-SOI-NW decreased with decreasing the concentration of the added target molecules from 3.3 × 10^−14^ M to 3.3 × 10^−17^ M. The concentration detection limit of oDVA in the measuring cell was *DL* = 10^−16^ M. After the oDVA solution was replaced with pure buffer, no significant change in the signal level was observed; this indicates slow dissociation of the complexes formed on the sensor surface.

## 4. Conclusions

Micro-Raman spectroscopy has been employed for monitoring the quality of h-k dielectric protective layer deposition on to the surface of SOI-NW structures with p-type conductivity. These structures were subsequently used for the fabrication of sensor chips intended for the highly sensitive detection of biological macromolecules. According to UV Raman spectroscopy data, a high density of ultra-dispersed HfO_2_ nanocrystals (obtained after annealing at >400 °C) with sizes smaller than the h-k dielectric thickness (10 nm) hinders them from further growth at higher annealing temperatures, also providing stability of properties of the h-k dielectrics stack in chemically and biologically active media. The application of such (h-k)-SOI-NW-based biosensors for the label-free real-time detection of oDVA with 10^−16^ M concentration sensitivity has been experimentally demonstrated, and better time stability of these sensor chips (in comparison with SOI-NW structures with native oxide) has been demonstrated. Taking into account that such (h-k)-SOI-NW chips provide high (*DL*~10^−16^ M) sensitivity of detection of DNA oligonucleotide analogue of miRNA-21, their application for the detection of miRNAs, associated with the early stages of cancer pathologies, is promising.
